# Local Net Charge State of Collagen Triple Helix Is a Determinant of FKBP22 Binding to Collagen III

**DOI:** 10.3390/ijms242015156

**Published:** 2023-10-13

**Authors:** Yoshihiro Ishikawa, Arkadiusz Bonna, Douglas B. Gould, Richard W. Farndale

**Affiliations:** 1 Department of Ophthalmology, University of California San Francisco, School of Medicine, San Francisco, CA 941583, USA; 2 Department of Biochemistry, Downing Site, Cambridge CB2 1QW, UK; 3 Department of Anatomy, University of California, San Francisco, CA 94143, USA; 4 Cardiovascular Research Institute, University of California, San Francisco, CA 94158, USA; 5 Bakar Aging Research Institute, University of California, San Francisco, CA 94143, USA; 6 Institute for Human Genetics, University of California, San Francisco, CA 94143, USA

**Keywords:** endoplasmic reticulum, extracellular matrix, Ehlers–Danlos Syndrome, collagen Toolkit, triple-helical peptides

## Abstract

Mutations in the *FKBP14* gene encoding the endoplasmic reticulum resident collagen-related proline isomerase FK506 binding protein 22 kDa (FKBP22) result in kyphoscoliotic Ehlers–Danlos Syndrome (EDS), which is characterized by a broad phenotypic outcome. A plausible explanation for this outcome is that FKBP22 participates in the biosynthesis of subsets of collagen types: FKBP22 selectively binds to collagens III, IV, VI, and X, but not to collagens I, II, V, and XI. However, these binding mechanisms have never been explored, and they may underpin EDS subtype heterogeneity. Here, we used collagen Toolkit peptide libraries to investigate binding specificity. We observed that FKBP22 binding was distributed along the collagen helix. Further, it (1) was higher on collagen III than collagen II peptides and it (2) was correlated with a positive peptide charge. These findings begin to elucidate the mechanism by which FKBP22 interacts with collagen.

## 1. Introduction

Collagens are one of the most abundant proteins in the human body [1] and are also among the most biosynthetically complex proteins [2,3,4,5]. Collagen biosynthesis is a highly orchestrated process involving over 20 proteins that takes place in the endoplasmic reticulum (ER). This complex biosynthetic machinery is collectively termed a molecular ensemble [6,7,8]. Mutations in genes encoding collagen cause many connective tissue disorders such as Osteogenesis Imperfecta (OI) and Ehlers–Danlos Syndrome (EDS), and their pathogenicity can be attributed to reduced levels of collagen proteins or the secretion of mutant collagen proteins [9,10,11,12,13]. Moreover, mutations in members of this molecular ensemble can also cause collagen-related connective tissue disorders, which have already been shown to be a cause of OI, establishing the paradigm of a genetic pathway extending from inside the cell to the extracellular matrix [14,15,16,17,18,19]. Similarly, in EDS, mutations in the *FKBP14* gene encoding FKBP22 (FK506 Binding Protein 22 kDa) result in kyphoscoliotic EDS [20,21,22,23,24,25]. FKBP22 acts as a molecular chaperone for type III collagen and catalyzes collagen folding through prolyl isomerase activity in the ER [26,27]. FKBP22 consists of a single FKBP domain, two EF-hand motifs, and an ER retention signal. Disruption of the EF-hand motifs has been found to change neither the prolyl isomerase activity nor the chaperone activity in vitro, suggesting that the FKBP domain is responsible for FKBP22 functions during collagen biosynthesis [26]. *FKBP14* mutations lead to loss of the FKBP22 protein usually due to mutations causing nonsense-mediated decay of the mRNA, and loss of FKBP22 causes a broad spectrum of clinical phenotypes, including progressive kyphoscoliosis, joint hypermobility, muscle hypotonia, hyperelastic skin, hearing loss, and aortic rupture [24,25]. The phenotypic spectrum may be explained by the selective binding of FKBP22 to some collagens (type III, IV, VI, and X) but not to others (type I, II, V, and XI) [26,27,28]. However, how and where FKBP22 differentially binds to collagen triple helices have never been explored. Therefore, in this report, we used collagen Toolkits, synthetic peptide libraries (CambCol Laboratories Ltd., Ely, UK) [29,30], to investigate the mechanism of FKBP22 recognition.

## 2. Results

### 2.1. Establishing a Method to Detect FKBP22 on the Collagen Toolkit Peptides

**Figure 1:**
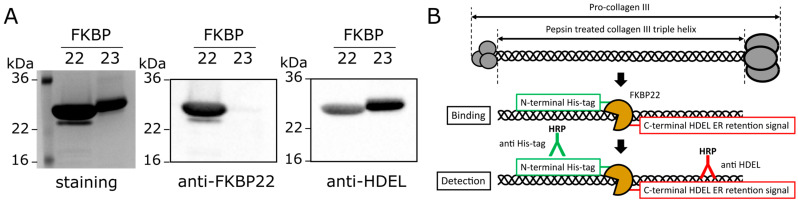
Establishing the detection method of the FKBP22 binding on collagen triple helices. (**A**). Western blotting analysis of purified recombinant human FKBP22 and FKBP23. The left image shows purified FKBPs running on a 16% Tris/Glycine gel under non-reducing condition stained with GelCode Blue Stain Reagent. The middle and right images show FKBPs were transferred to a PVDF membrane and subsequently analyzed by western blotting using antibodies against FKBP22 and HDEL. The uncropped images used in (**A**) are presented in Supplemental Figure S1. (**B**) Diagram representing the strategy used to detect the FKBP22 binding on collagen triple helices by HRP-conjugated antibodies.

FKBP22 binding to collagens was determined by direct protein–protein binding measurements with surface plasmon resonance (SPR) using un-tagged FKBP22 [26]. In contrast, for FKBP22 binding to the collagen Toolkits, we used an ELISA-like assay that requires indirect detection by antibody and commonly uses the biochemical tags V5, CaM and 6xHistidine (His-) for detection. We first tested if a carboxyl terminal ER retention signal HDEL might be suitable for detection [31]. We expressed and purified recombinant human FKBP22 with an amino terminal His-tag (His-FKBP22) to validate the detection using HRP-conjugated antibodies against His-tag and HDEL in the ELISA-like binding assay. We confirmed by western blotting that this antibody recognized an HDEL sequence in both FKBP22 and FKBP23, which similarly has a carboxyl terminal HDEL ER retention signal [32,33] (Figure 1A and Figure S1).

Next, we applied His-FKBP22 to immobilized, purified, pepsin-treated, full-length collagen III to assess the detection efficiency using the HRP-conjugated antibodies against His-tag and HDEL (Figure 1B). The ELISA-like assay showed that the His-tag antibody was able to detect the FKBP22 bound to collagen III in a concentration-dependent manner (Table 1). Conversely, we were unable to detect FKBP22 binding to collagen III using the HDEL antibody (Table 1). These results suggested that the un-tagged FKBP22 used for SPR was not acceptable for the collagen Toolkit peptides. Thus, His-FKBP22 and the His-tag antibody were used for further investigations.

The baseline signals without His-FKBP22 were subtracted from the signal with His-FKBP22. Values in the table are presented as means ± S.D. There were three replicate experiments for each condition.

### 2.2. The Collagen Toolkit III Captured Significantly More FKBP22 Than the Collagen Toolkit II

**Figure 2:**
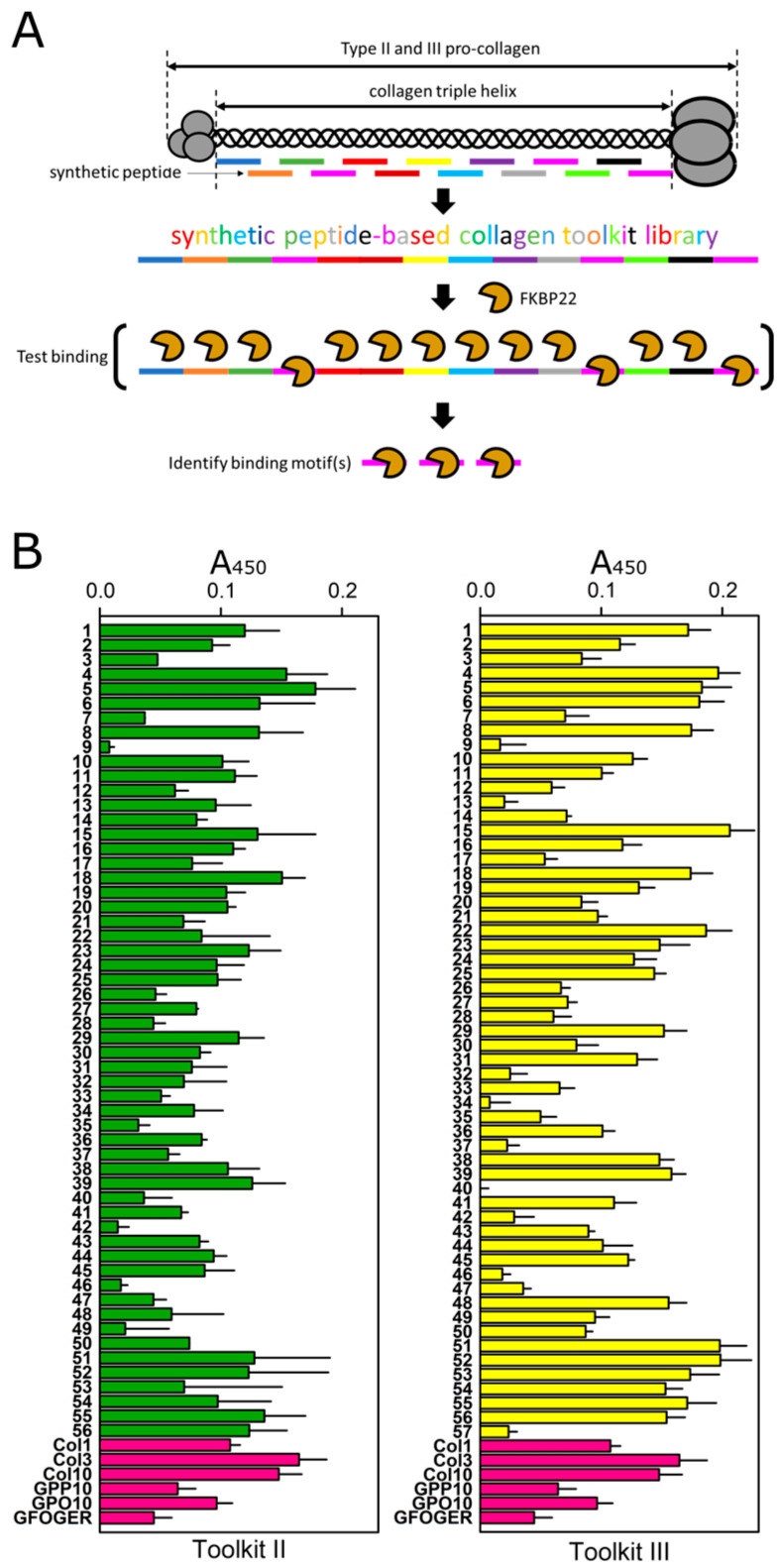
Identification of the FKBP22 binding motif(s) on collagen triple helices. (**A**) Diagram representing the strategy used to identify the FKBP22 binding motif(s) on collagen triple helices. (**B**) ELISA-like binding assays to identify the peptides recognized by recombinant FKBP22 (25 µM) using the collagen II and III synthetic peptide Toolkits. The green and yellow bars show binding to Toolkit II and III (coated with 1.0 µg/well to 96 well plates), respectively. The magenta bars show control peptides coated to 96 well plates: GPP10, GPO10 and integrin-binding peptide GFOGER (1.0 µg/well). Collagen I (Col1: 5.0 µg/well) was used as a negative control, and collagens III (Col3: 5.0 µg/well) and X (Col10: 2.0 µg/well) were used as positive controls. Signals are reported as absorbance at 450 nm (A_450_). In each assay, the five lowest signals were averaged and used as the baseline to be subtracted from other signals. Values in the graphs are presented as means ± S.E. The number of biological replicates was n = 3 for each Toolkit.

Since FKBP22 binds to type III but not type II collagen [26], we applied His-FKBP22 to immobilized collagen III Toolkit (Toolkit III) peptides to identify the FKBP22 binding motif(s), and we used collagen II Toolkit (Toolkit II) peptides as negative controls (Figure 2A) in solid-phase ELISA-like binding assays. In each ELISA-like binding assay, we included purified collagen III and X or purified collagen I as internal positive and negative controls, respectively. We identified some degree of His-FKBP22 binding to almost all the Toolkit II and III peptides (Figure 2B).

**Figure 3:**
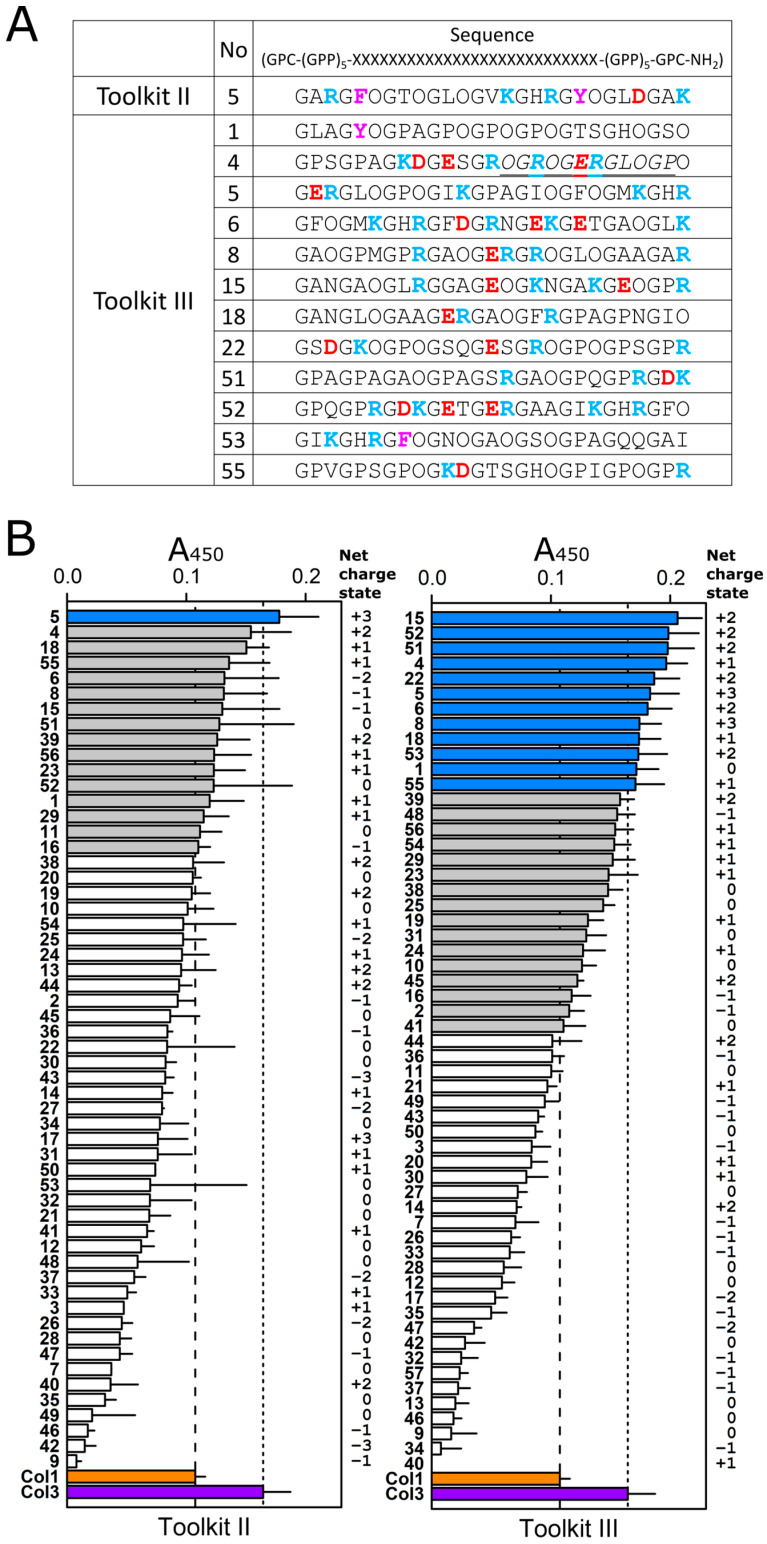
Collagen Toolkit III captured significantly more FKBP22 than collagen Toolkit II. (**A**) The strong affinity peptide sequences. O: 4-hydroxyproline; Blue: positive charge; Red: negative charge; Purple: aromatic residue; italics with underline: sequence appears in collagen X. (**B**) Signals in Figure 1B are reordered from high to low with thresholds indicated for collagen I (Col1 orange bar) and collagen III (Col3 purple bar). White and blue bars indicate peptides below the Col1 threshold and above the Col3 threshold, respectively. Gray bars indicate peptides between Col1 and Col3 threshold. The left side of each panel shows peptide numbers, and the right side indicates the net charge state calculated by the sum of positively charged amino acids (arginine (R) and lysine (K)) and negatively charged amino acids (aspartate (D) and glutamate (E)).

Strong affinity FKBP22-binding peptides were defined as A_450_ > 0.16 based on the positive control collagen III. We found that Toolkit III captured significantly more His-FKBP22 than Toolkit II (Figure 3A,B), which corresponds to the findings of the previous report [26]. Using binding sequence(s) empirically defined from these data, we sought to find similar sequences in collagen X, which is also homotrimeric and has been shown to be a putative FKBP22 substrate [26,27]. The sequence OGROGERGLOGP (O: 4Hyp) in the Toolkit III peptide 4 was found to correspond to amino acids P195 to P207 (counted from the first methionine) in the human and mouse collagen X sequence (UniProt entries Q03692 and Q05306, respectively; Figure 3A), and we suggest that FKBP22 recognizes this region in collagen X.

### 2.3. Local Net Charge State Contributes to the Binding of FKBP22 to Collagen Toolkit III Peptides

**Figure 4:**
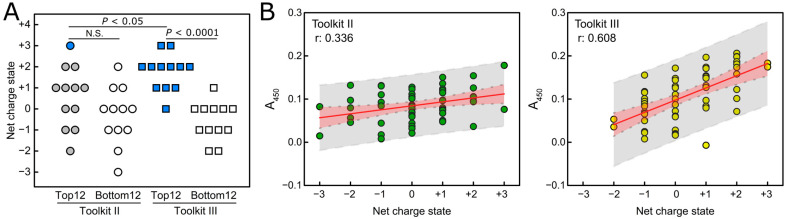
Net charge state of collagen peptides plays an important role in FKBP22 binding. (**A**) The top and bottom 12 are the 12 highest and 12 lowest FKBP22-binding peptides, respectively, among Toolkits II and III peptides. Binding levels to these peptides were plotted versus net charge, as shown in Figure 2B. The *p* values were determined by the Mann–Whitney test. N.S. indicates ‘no significance’. Dot colors correspond with those described in Figure 3B. (**B**) The binding signals were plotted against peptide net charge, and the relationship between them was measured by simple linear regression analysis. The estimated regression line and the Pearson correlation coefficient are shown as a red line and r, respectively. Red and gray bands indicate the 95% confidence and 95% prediction intervals, respectively.

We predicted that we would identify a specific FKBP22 binding sequence from Toolkit III, as has been previously demonstrated for von Willebrand factor, matrix metalloproteinases, integrins, and small leucine-rich repeat proteoglycans using the same methods [30]. However, this binding activity was widespread and more similar in this respect to that observed for a collagen-binding bacterial adhesin, YadA [34]. The binding profile of YadA to Toolkit II resembled that of Toolkit III [34], while the binding profile of FKBP22 differed between Toolkits II and III (Figure 3B). This suggests that FKBP22 recognizes collagen peptides using some set of rules. Here, we propose a role for the net charge state of each Toolkit peptide, which is calculated by the sum of the positively charged amino acids, Arginine (R) and Lysine (K), and the negatively charged amino acids, aspartate (D) and glutamate (E) (Figure 3A,B). Interestingly, we found that most of the higher-affinity FKBP22-binding peptides in both Toolkits II and III carried a positive net charge (Figure 3B). Therefore, we compared the charge of the 12 highest and 12 lowest FKBP22-binding peptides within each Toolkit using the non-parametric Mann–Whitney test. This revealed a significant difference between the two groups (*p* < 0.0001) for Toolkit III but not Toolkit II, suggesting that the net charge state contributes to the binding of FKBP22 to Toolkit III peptides (Figure 4A). Moreover, simple linear regression analysis showed that overall FKBP22 binding capability was more strongly correlated with the net charge of the collagen peptides in Toolkit III than in Toolkit II (Figure 4B). Some peptides were found to be anomalous: TK-III-1 (Toolkit III peptide 1), for example, had a high affinity for FKB22 but was uncharged (Figure 3A,B), suggesting that other interactions may also be important. Taken together, although it is not sufficient to explain the entire mechanism of FKBP22 binding to specific collagen types, charge clearly plays an important role in this binding.

## 3. Discussion

A previous direct binding study using SPR demonstrated that FKBP22 did not interact with immobilized, purified, pepsin-treated, full-length collagen II [26]. Here, we observed that some Toolkit II peptides captured His-FKBP22, although most of these peptides interacted weakly. However, overall, Toolkit III peptides were found to bind more strongly than those from Toolkit II (Figure 3A,B).

We identified FKBP22-binding peptides from the Toolkits, and analysis of these suggested two important conclusions: (i) that the local charge state of the collagen triple helix is critical for FKBP22 recognition, providing a potential mechanism for its recognition by FKBP22 (Figure 3 and Figure 4), and (ii) that collagen binding is defined by other aspects of sequence specificity than charge alone.

The ranked binding data (Figure 3B) indicate that charge alone is not sufficient to define the ability to bind collagen peptides. Eight peptides were found in Toolkit II with a charge of either +2 or +3, which did not exceed our threshold binding activity, compared with just four in Toolkit III. Meanwhile, two peptides in both toolkits fell between the Col1 and Col3 signal threshold, and six and two peptides were below the Col1 threshold in Toolkits II and III, respectively (Figure 3B). We speculate that collagen triple helices must have a unique local amino acid composition that complements the FKBP22-preferred positive net charge state resulting from the combination of amino acids R/K and D/E. However, we are unable to define a conserved sequence such as the GXXGER motif identified for integrin–collagen binding [30]. However, although we only tested collagen III, we found a potential sequence in collagen X (Figure 2A), which is also a homotrimeric collagen.

It is important to consider how surface charge may be organized in heterotrimeric collagens such as collagen IV and VI. Further studies are required to establish the detailed binding mechanism of FKBP22 with high affinity peptides and for chain recognition in the collagen triple helix. Higher affinity peptides frequently occur in tandem in the N- and C-terminal regions of collagen III (Figure 2B). Examples of this include TK-III-5 and -6, and TK-III-52 and -53. Each of these peptides carries a net positive charge and includes a 9-amino acid overlap sequence that contains the positive tract, KGHR (charge +2), which is a crosslinking motif [30]. Interestingly, KGHR also occurs in the lower-binding TK-II-6 and TK-II-52 and -53, but each of these carries a net negative or neutral charge, whereas the corresponding TK-II-5 is both high-binding and positively charged. For binding FKBP22, this clearly indicates the importance of overall charge rather than specific positively charged motifs in the collagen triple helix.

Heat shock protein 47 kDa (HSP47), like FKBP22, is a collagen molecular chaperone [15], and its mechanism of binding to collagen is well-established [35,36,37]. Histidine residues in the HSP47 sequence have been identified in the binding interface between canine HSP47 and collagen model peptides, and have been suggested to regulate the binding of HSP47 to collagens [38,39], driven by protonation in the lower pH of the Golgi apparatus rather than that of the ER, with consequent dissociation of the HSP47 occurring from a complex with collagen that permits collagen secretion [40,41]. Although histidine has a basic side chain, its pK_a_ in a short peptide has been estimated to be about 6.8 [42]. Hence, at pH 7.4 (the pH of both the ER and our assay), histidine is likely to be predominantly neutral, unlike lysine and arginine, both of which are fully protonated. These considerations apply to histidine residues in FKBP22 as well as HSP47. In this context, the amino terminal His-tag in FKBP22 is unlikely to contribute to charge-mediated interactions with Toolkit peptides for the same reason: the His-tag carries close to a neutral charge at the pH used in our studies and will become electropositive if it coordinates calcium, which is contained in our ELISA-like binding assays.

More than 500 mutations in *COL3A1* have been reported to cause vascular EDS (OMIM # 130050) [43]. Collagen triple helix formation proceeds from the C- to the N-terminal end, and mutations nearer the C-terminus are generally associated with more severe pathology [44]. Indeed, although a study found that glycine substitutions of centrally located G499D and G415S residues showed slower SDS-PAGE gel migration (over-modification) and impaired secretion, respectively [45,46], glycine substitution at a more N-terminal residue (G130R) appeared normal in SDS-PAGE gel migration [47]. G130 is close to a potential integrin α1β1-binding site, GLOGEN [30], and the inclusion of collagen III with G130R in the extracellular matrix might explain this deleterious effect. However, we note that as G130 is in peptide TK-III-8 (charge +3), which shows a strong binding affinity, this glycine substitution would result in a disruption of local helix stability. Hence, deficient FKBP22 binding around G130 could be another potential explanation for the clinical phenotype of vascular EDS in addition to an integrin binding deficiency.

Our results raise the following question: how does FKBP22 recognize the positively charged surface of the collagen triple helix? FKBP22 consists of a single FKBP domain, two EF-hand motifs, and an ER retention signal [26]. In vitro studies indicate that the FKBP domain isomerizes proline residues, showing chaperone activity involved in collagen biosynthesis, and interacts with collagen independently of the EF-hand motifs [26,27]. The crystal structure of FKBP22 is solved, and negatively charged regions exist within each EF-hand motif [27,48], as well as elsewhere in FKBP22. Moreover, electron density analysis indicates that the second EF-hand motif has a unique surface environment for coordinating metal ions [48]. The recruitment of divalent cations to the second EF-hand in the cation-rich environment of the ER would render these regions of FKBP22 more positive, tending to repel electropositive tracts of collagen, so it seems unlikely that this domain contains the collagen-binding site. The first EF-hand motif appears to be a better candidate binding site.

We show in Table 1 that the anti-HDEL antibody detected FKBP22 very poorly when the latter was bound to collagen III, whereas detection was promising in the western blotting. This suggests that accessibility to HDEL may be restricted by collagen binding, possibly because HDEL lies in or near to the collagen-binding site in FKBP22 and that collagen engagement obscures the epitopes, or alternatively, that the antibody binds successfully to HDEL, releasing FKBP22 from collagen. We speculate that the strong interaction between HDEL and the anti-HDEL antibody might alter the binding status of His-FKBP22 to collagen III, since the binding kinetics (on and off rate) have been found to be very fast [26]. The idea that HDEL forms part of the collagen-binding site is quite attractive: HDEL is electronegative, which may contribute to the interaction of FKBP22 with electropositive tracts of collagen. Moreover, as the complex moves towards the Golgi apparatus, HDEL becomes less electronegative due to the protonation of its histidine residue at lower pH. This effect might release collagen from FKBP22 in the same manner proposed above for HSP47.

In summary, our study successfully generated a map of the recognition sequences in collagen III and predicted the importance of local net charge for the binding of FKB22 to the collagen triple helix, paving the way for many other potential studies that will help define this interaction. We anticipate that the EF-hand motifs, particularly the HDEL sequence, may contribute to the FKBP22–collagen interaction, and may act as a hub to recognize the positively charged surface of the collagen triple helix and enhance the functional efficiency of the FKBP domain of FKBP22. Further biochemical studies and molecular dynamics simulations as a complement [49] should be performed to test this hypothesis using un-tagged (native) FKBP22 with one or two of the best binding peptides.

## 4. Materials and Methods

### 4.1. Preparation of His-FKBP22 and FKBP23

Human FKBP22 and FKBP23 were constructed as described previously [26,31]. The expression vectors were transformed into *E. coli* BL21 (DE3) and grown at 37  °C to A_600_ = 0.6, and expression was induced with 1 mM isopropyl β-D-1-thiogalactopyranoside. After incubation at 20  °C overnight, the cells were harvested by centrifugation and resuspended in Tris base B-PER (Thermo Scientific, Waltham, MA, USA) containing 1 mM CaCl_2_. Insoluble material was removed by centrifugation, and proteins in the soluble fraction were precipitated with ammonium sulfate at a final concentration of 30% (*w*/*v*). After overnight incubation at 4 °C, the sample was centrifuged, and the precipitated materials were dissolved in HEPES buffer (20 mM HEPES buffer, pH 7.5, containing 1.0 M NaCl, 20 mM imidazole, and 1 mM CaCl_2_). The protein solution was passed through a 0.22-μm filter and loaded onto a Co^2+^-chelating column. After washing with HEPES buffer (minimum 5 column volumes), FKBP22 and FKBP23 with the amino terminal His-tag were eluted with elution buffer (20 mM HEPES buffer, pH 7.5, containing 1.0 M NaCl, 500 mM imidazole, and 1 mM CaCl_2_). The fractions containing these FKBPs were dialyzed against HBS (10 mM HEPES, pH 7.4, containing 0.15 M NaCl and 1 mM CaCl_2_) for western blotting analysis and the ELISA assays.

### 4.2. Preparation of Collagens

The preparation of purified, pepsin-treated, full-length collagens I and III has been described previously [26]. Briefly, collagen I and III extraction was performed using adult murine skin. All procedures were performed at 4 °C. Pieces of skin were dissolved into excess volumes of 0.5 M acetic acid and incubated for several hours. Pepsin (Sigma Aldrich, St. Louis, MO, USA) was added to a final concentration of 0.1 mg/mL, and tissues were digested at 4 °C overnight. The solutions were centrifuged to remove insoluble material, and then NaCl was added to a final concentration of 0.7 M to precipitate collagens. The solution was then incubated overnight at 4 °C. Precipitates were collected by centrifugation at 13,000 rpm for 30 min using JA-14 rotor (Beckman, Brea, CA, USA) and then resuspended in 0.2 M acetic acid. This collagen solution was dialyzed to 0.1 M Tris/HCl containing 1.0 M NaCl, and at pH 7.5, overnight at 4 °C. NaCl was added to a final concentration of 1.6 M to preferentially precipitate collagen III. After incubation overnight at 4 °C, collagen III was collected by centrifugation at 13,000 rpm for 30 min using JA-20 rotor (Beckman), and the supernatant was found to contain enriched collagen I. Collagen I was precipitated by centrifugation at 13,000 rpm for 30 min using JA-20 rotor after adding NaCl to a final concentration of 2.4 M and incubating this overnight at 4 °C. The pellets were resolubilized in 0.2 M acetic acid and dialyzed to the same concentration of acetic acid overnight at 4 °C to remove residual NaCl for experiments. The preparation of purified full-length collagens X has been described previously [50].

### 4.3. Western Blotting Analysis

Purified FKBP22 and FKBP23 were desaturated with 4X Bolt LDS sample buffer (N) Thermo Scientific, Waltham, MA, USA) without reducing agents. These proteins were separated on Novex WedgeWell 16% Tris-Glycine gel (Thermo Fisher Scientific) and then stained with GelCode Blue Stain Reagent (Thermo Fisher Scientific) or transferred to PVDF membranes (Bio-Rad, South Granville, NSW, Australia). Western blots were performed with the PVDF membranes using FKBP22 Polyclonal antibody (ProteinTech: 15884-1-AP, Rosemont, IL, USA) and HRP-conjugated HDEL Monoclonal antibody (Santa Cruz Biothechnology: sc-5472 HRP, Dallas, TX, USA). Blots were developed with horseradish-peroxidase-enhanced Super-Signal West Pico Chemiluminescent Substrate (Thermo Fisher Scientific) and detected by ChemiDoc MP imaging system (Bio-Rad) using the software Image Lab, version 4.0.1 (Bio-Rad).

### 4.4. ELISA-like Binding Assay with Purified Pepsin Treated Collagen III

Purified pepsin treated collagen III was coated onto a 96 well plate at 5.0 µg/well in 20 mM acetic acid overnight at 4 °C. Plates were rinsed three times with HBS and blocked with 5% BSA in HBST (10 mM HEPES, pH 7.4, containing 0.15 M NaCl, 1 mM CaCl_2_ and 0.05% tween 20) for 1 h at room temperature. Plates were rinsed three times with HBS and incubated with 10 or 40 µM His-FKBP22 overnight at 4 °C. After plates were rinsed three times with HBS, HRP-conjugated 6xHistidine tag Monoclonal antibody (Proteintech: HRP-66005) and HRP-conjugated HDEL Monoclonal antibody were added at 1:1000 dilution in HBS for 1 h at room temperature. After washing with HBS three times, the binding was detected with SuperSignal ELISA Femto Substrate (Thermo Scientific: 37075) according to supplier’s instructions, and A_450_ was recorded.

### 4.5. Collagen Toolkit Peptides

Collagen II and III Toolkit peptides were purchased from CambCol Laboratories Ltd., UK. The sequences of peptides used for coating are listed in Table S1.

### 4.6. ELISA-like Binding Assay with Collagen Toolkit Peptides

Each assay included His-FKBP22, full-length collagens III and X as positive controls and BSA and full-length collagen I as negative controls. Toolkit peptides were coated at 10 µg/mL in 20 mM acetic acid overnight at 4 °C. Plates were rinsed three times with HBS and blocked with 5% BSA in HBST (10 mM HEPES, pH 7.4, containing 0.15 M NaCl, 1 mM CaCl_2_ and 0.05% tween 20) for 1 h at room temperature. Plates were rinsed three times with HBS and incubated with 25 µM His-FKBP22 overnight at 4 °C. After plates were rinsed three times with HBS, HRP-conjugated 6xHis-tag Monoclonal antibody (Proteintech: HRP-66005) was added at 1:1000 dilution in HBS for 1 h at room temperature. After washing with HBS three times, the binding was detected with SuperSignal ELISA Femto Substrate (Thermo Scientific: 37075) according to supplier’s instructions, and A_450_ was recorded.

### 4.7. Statistical and Plotting Analyses

For comparisons between two groups, the Mann–Whitney test was performed to determine whether differences between groups were significant. A *p* value of less than 0.05 was considered statistically significant. To estimate the relationship between two dependent variables, simple linear regression analysis was performed. OriginPro, version 9.1 (OriginLab Corp. Northampton, MA, USA) was used for these analyses. We eliminated TK-III-57 for these analyses because its length is three amino acids shorter than others.

## Figures and Tables

**Table 1:** ELISA-like binding assays to detect the binding of His-FKBP22 to collagen III using two different antibodies.

Detection	His-Tag Antibody		HDEL Antibody	
His-FKBP22 (µM)	10	40	10	40
A_450_	0.137 ± 0.092	0.328 ± 0.04	0.013 ± 0.005	0.043 ± 0.014

## Data Availability

All data in this publication are available from the corresponding author upon reasonable request.

## Supplementary Materials

The supporting information can be downloaded at: https://www.mdpi.com/article/10.3390/ijms242015156/s1.
