# Supplementary material for: Local Net Charge State of Collagen Triple Helix Is a Determinant of FKBP22 Binding to Collagen III
Source: Int J Mol Sci. 2023 Oct 13;24(20):15156. doi: 10.3390/ijms242015156 (PMC10607241; doi:10.3390/ijms242015156)
Supplement: Supplementary file 1 [file ijms-24-15156-s001.zip › ijms-2598764-supplementary.pdf]

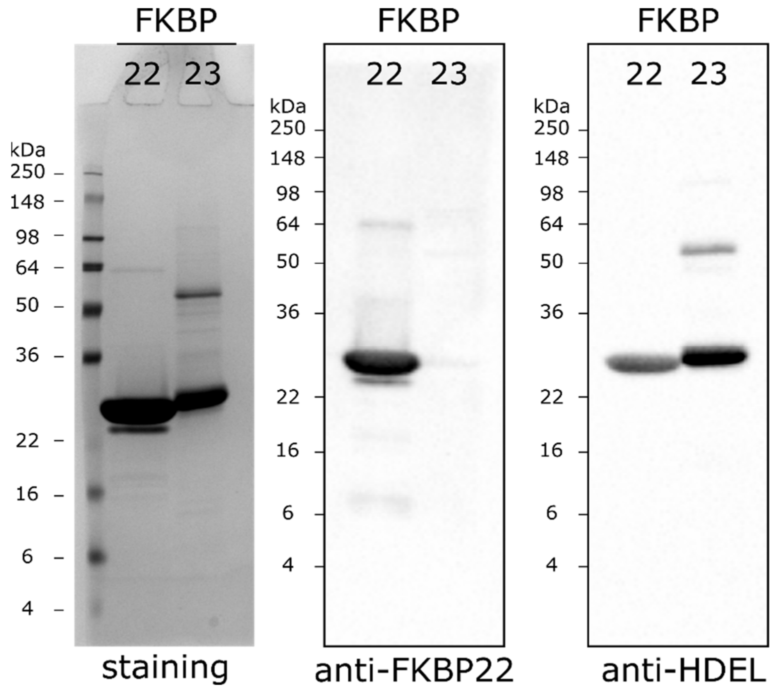

Figure S1. Uncropped images of GelCode Blue Stained SDS/PAGE gel and Western blots presented in Figure 1B

Table S1. The sequences of Collagen II and III Toolkit peptides used for coating on a 96 well plate.

Collagen II Toolkit Peptides		Collagen III Toolkit Peptides	
1	GPC-(GPP)$_5$-GPMGPMGPRGPOGPAGAOGPQGFQGNO-(GPP)$_5$-GPC-NH_2	1	GPC-(GPP)$_5$-GLAGYOGPAGPOGPOGPOGTSGHOGSO-(GPP)$_5$-GPC-NH_2
2	GPC-(GPP)$_5$-GPQGFQGNOGEOGEOGVSGPMGPRGPO-(GPP)$_5$-GPC-NH_2	2	GPC-(GPP)$_5$-GTSGHOGSOGSOGYQGPOGEOGQAGPS-(GPP)$_5$-GPC-NH_2
3	GPC-(GPP)$_5$-GPMGPRGPOGPOGKOGDDGEAGKOGKA-(GPP)$_5$-GPC-NH_2	3	GPC-(GPP)$_5$-GEOGQAGPSGPOGPOGAIGPSGPAGKD-(GPP)$_5$-GPC-NH_2
4	GPC-(GPP)$_5$-GEAGKOGKAGERGPOGPQGARGFOGTO-(GPP)$_5$-GPC-NH_2	4	GPC-(GPP)$_5$-GPSGPAGKDGESGROGROGERGLOGPO-(GPP)$_5$-GPC-NH_2
5	GPC-(GPP)$_5$-GARGFOGTOGLOGVKGHRGYOGLDGAK-(GPP)$_5$-GPC-NH_2	5	GPC-(GPP)$_5$-GERGLOGPOGIKGPAGIOGFOGMKGHR-(GPP)$_5$-GPC-NH_2
6	GPC-(GPP)$_5$-GYOGLDGAKGEAGAOGVKGESGSOGEN-(GPP)$_5$-GPC-NH_2	6	GPC-(GPP)$_5$-GFOGMKGHRGFDGRNGEKGETGAOGLK-(GPP)$_5$-GPC-NH_2
7	GPC-(GPP)$_5$-GESGSOGENGSOGPMGPRGLOGERGRT-(GPP)$_5$-GPC-NH_2	7	GPC-(GPP)$_5$-GETGAOGLKGENGLOGENGAOGPMGPR-(GPP)$_5$-GPC-NH_2
8	GPC-(GPP)$_5$-GLOGERGRTGPAGAAGARGNDGQOGPA-(GPP)$_5$-GPC-NH_2	8	GPC-(GPP)$_5$-GAOGPMGPRGAOGERGROGLOGAAGAR-(GPP)$_5$-GPC-NH_2
9	GPC-(GPP)$_5$-GNDGQOGPAGPOGPVGPAGGOGFOGAO-(GPP)$_5$-GPC-NH_2	9	GPC-(GPP)$_5$-GLOGAAGARGNDGARGSDGQOGPOGPO-(GPP)$_5$-GPC-NH_2
10	GPC-(GPP)$_5$-GGOGFOGAOGAKGEAGPTGARGPEGAQ-(GPP)$_5$-GPC-NH_2	10	GPC-(GPP)$_5$-GQPGPOGPOGTAGFOGSOGAKGEVGPA-(GPP)$_5$-GPC-NH_2
11	GPC-(GPP)$_5$-GARGPEGAQGPRGEOGTOGSOGPAGAS-(GPP)$_5$-GPC-NH_2	11	GPC-(GPP)$_5$-GAKGEVGPAGSOGSNGAOGQRGEOGPQ-(GPP)$_5$-GPC-NH_2
12	GPC-(GPP)$_5$-GSOGPAGASGNOGTDGIOGAKGSAGAO-(GPP)$_5$-GPC-NH_2	12	GPC-(GPP)$_5$-GQRGEOGPQGHAGAQGPOGPOGINGSO-(GPP)$_5$-GPC-NH_2
13	GPC-(GPP)$_5$-GAKGSAGAOGIAGAOGFOGPRGPOGPQ-(GPP)$_5$-GPC-NH_2	13	GPC-(GPP)$_5$-GPOGINGSOGGKGEMGPAGIOGAOGLM-(GPP)$_5$-GPC-NH_2
14	GPC-(GPP)$_5$-GPRGPOGPQGATGPLGPKGQTGEOGIA-(GPP)$_5$-GPC-NH_2	14	GPC-(GPP)$_5$-GIOGAOGLMGARGPOGPAGANGAOGLR-(GPP)$_5$-GPC-NH_2
15	GPC-(GPP)$_5$-GQTGEOGIAGFKGEQGPKGEOGPAGPQ-(GPP)$_5$-GPC-NH_2	15	GPC-(GPP)$_5$-GANGAOGLRGGAGEOGKNGAKGEOGPR-(GPP)$_5$-GPC-NH_2
16	GPC-(GPP)$_5$-GEOGPAGPQGAOGPAGEEGKRGARGEO-(GPP)$_5$-GPC-NH_2	16	GPC-(GPP)$_5$-GAKGEOGPRGERGEAGIOGVOGAKGED-(GPP)$_5$-GPC-NH_2
17	GPC-(GPP)$_5$-GKRGARGEOGGVGPIGPOGERGAOGNR-(GPP)$_5$-GPC-NH_2	17	GPC-(GPP)$_5$-GVOGAKGEDGKDGSOGEOGANGLOGAA-(GPP)$_5$-GPC-NH_2
18	GPC-(GPP)$_5$-GERGAOGNRGFOGQDGLAGPKGAOGER-(GPP)$_5$-GPC-NH_2	18	GPC-(GPP)$_5$-GANGLOGAAGERGAOGFRGPAGPNGIO-(GPP)$_5$-GPC-NH_2
19	GPC-(GPP)$_5$-GPKGAOGERGPSGLAGPKGANGDOGRO-(GPP)$_5$-GPC-NH_2	19	GPC-(GPP)$_5$-GPAGPNGIOGEKGPAGERGAOGPAGPR-(GPP)$_5$-GPC-NH_2
20	GPC-(GPP)$_5$-GANGDOGROGEOGLOGARGLTGROGDA-(GPP)$_5$-GPC-NH_2	20	GPC-(GPP)$_5$-GAOGPAGPRGAAGEOGRDGVOGGOGMR-(GPP)$_5$-GPC-NH_2
21	GPC-(GPP)$_5$-GLTGROGDAGPQGKVGPSGAOGEDGRO-(GPP)$_5$-GPC-NH_2	21	GPC-(GPP)$_5$-GVOGGOGMRGMOGSOGGOGSDGKOGPO-(GPP)$_5$-GPC-NH_2
22	GPC-(GPP)$_5$-GAOGEDGROGPOGPQGARGQOGVMGFO-(GPP)$_5$-GPC-NH_2	22	GPC-(GPP)$_5$-GSDGKOGPOGSQGESGROGPOGPSGPR-(GPP)$_5$-GPC-NH_2
23	GPC-(GPP)$_5$-GQOGVMGFOGPKGANGEOGKAGEKGLO-(GPP)$_5$-GPC-NH_2	23	GPC-(GPP)$_5$-GPOGPSGPRGQOGVMGFOGPKGNDGAO-(GPP)$_5$-GPC-NH_2
24	GPC-(GPP)$_5$-GKAGEKGLOGAOGLRGLOGKDGETGAA-(GPP)$_5$-GPC-NH_2	24	GPC-(GPP)$_5$-GPKGNDGAOGKNGERGGOGGOGPQGPO-(GPP)$_5$-GPC-NH_2
25	GPC-(GPP)$_5$-GKDGETGAAGPOGPAGPAGERGEQGAO-(GPP)$_5$-GPC-NH_2	25	GPC-(GPP)$_5$-GGOGPQGPOGKNGETGPQGPOGPTGPG-(GPP)$_5$-GPC-NH_2
26	GPC-(GPP)$_5$-GERGEQGAOGPSGFQGLOGPOGPOGEG-(GPP)$_5$-GPC-NH_2	26	GPC-(GPP)$_5$-GPOGPTGPGGDKGDTGPOGPQGLQGLO-(GPP)$_5$-GPC-NH_2
27	GPC-(GPP)$_5$-GPOGPOGEGGKOGDQGVOGEAGAOGLV-(GPP)$_5$-GPC-NH_2	27	GPC-(GPP)$_5$-GPQGLQGLOGTGGPOGENGKOGEOGPK-(GPP)$_5$-GPC-NH_2
28	GPC-(GPP)$_5$-GEAGAOGLVGPRGERGFOGERGSOGAQ-(GPP)$_5$-GPC-NH_2	28	GPC-(GPP)$_5$-GKOGEOGPKGDAGAOGAOGGKGDAGAO-(GPP)$_5$-GPC-NH_2
29	GPC-(GPP)$_5$-GERGSOGAQGLQGPRGLOGTOGTDGPK-(GPP)$_5$-GPC-NH_2	29	GPC-(GPP)$_5$-GGKGDAGAOGERGPOGLAGAOGLRGGA-(GPP)$_5$-GPC-NH_2
30	GPC-(GPP)$_5$-GTOGTDGPKGASGPAGPOGAQGPOGLQ-(GPP)$_5$-GPC-NH_2	30	GPC-(GPP)$_5$-GAOGLRGGAGPOGPEGGKGAAGPOGPO-(GPP)$_5$-GPC-NH_2
31	GPC-(GPP)$_5$-GAQGPOGLQGMOGERGAAGIAGPKGDR-(GPP)$_5$-GPC-NH_2	31	GPC-(GPP)$_5$-GAAGPOGPOGAAGTOGLQGMOGERGGL-(GPP)$_5$-GPC-NH_2
32	GPC-(GPP)$_5$-GIAGPKGDRGDVGEKGPEGAOGKDGGR-(GPP)$_5$-GPC-NH_2	32	GPC-(GPP)$_5$-GMOGERGGLGSOGPKGDKGEOGGOGAD-(GPP)$_5$-GPC-NH_2
33	GPC-(GPP)$_5$-GAOGKDGGRGLTGPIGPOGPAGANGEK-(GPP)$_5$-GPC-NH_2	33	GPC-(GPP)$_5$-GEOGGOGADGVOGKDGPRGPTGPIGPO-(GPP)$_5$-GPC-NH_2
34	GPC-(GPP)$_5$-GPAGANGEKGEVGPOGPAGSAGARGAO-(GPP)$_5$-GPC-NH_2	34	GPC-(GPP)$_5$-GPTGPIGPOGPAGQOGDKGEGGAOGLO-(GPP)$_5$-GPC-NH_2
35	GPC-(GPP)$_5$-GSAGARGAOGERGETGPOGPAGFAGPO-(GPP)$_5$-GPC-NH_2	35	GPC-(GPP)$_5$-GEGGAOGLOGIAGPRGSOGERGETGPO-(GPP)$_5$-GPC-NH_2
36	GPC-(GPP)$_5$-GPAGFAGPOGADGQOGAKGEQGEAGQK-(GPP)$_5$-GPC-NH_2	36	GPC-(GPP)$_5$-GERGETGPOGPAGFOGAOGQNGEOGGK-(GPP)$_5$-GPC-NH_2
37	GPC-(GPP)$_5$-GEQGEAGQKGDAGAOGPQGPSGAOGPQ-(GPP)$_5$-GPC-NH_2	37	GPC-(GPP)$_5$-GQNGEOGGKGERGAOGEKGEGGPOGVA-(GPP)$_5$-GPC-NH_2
38	GPC-(GPP)$_5$-GPSGAOGPQGPTGVTGPKGARGAQGPO-(GPP)$_5$-GPC-NH_2	38	GPC-(GPP)$_5$-GEGGPOGVAGPOGGSGPAGPOGPQGVK-(GPP)$_5$-GPC-NH_2
39	GPC-(GPP)$_5$-GARGAQGPOGATGFOGAAGRVGPOGSN-(GPP)$_5$-GPC-NH_2	39	GPC-(GPP)$_5$-GPOGPQGVKGERGSOGGOGAAGFOGAR-(GPP)$_5$-GPC-NH_2
40	GPC-(GPP)$_5$-GRVGPOGSNGNOGPOGPOGPSGKDGPK-(GPP)$_5$-GPC-NH_2	40	GPC-(GPP)$_5$-GAAGFOGARGLOGPOGSNGNOGPOGPS-(GPP)$_5$-GPC-NH_2
41	GPC-(GPP)$_5$-GPSGKDGPKGARGDSGPOGRAGEOGLQ-(GPP)$_5$-GPC-NH_2	41	GPC-(GPP)$_5$-GNOGPOGPSGSOGKDGPOGPAGNTGAO-(GPP)$_5$-GPC-NH_2
42	GPC-(GPP)$_5$-GRAGEOGLQGPAGPOGEKGEOGDDGPS-(GPP)$_5$-GPC-NH_2	42	GPC-(GPP)$_5$-GPAGNTGAOGSOGVSGPKGDAGQOGEK-(GPP)$_5$-GPC-NH_2
43	GPC-(GPP)$_5$-GEOGDDGPSGAEGPOGPQGLAGQRGIV-(GPP)$_5$-GPC-NH_2	43	GPC-(GPP)$_5$-GDAGQOGEKGSOGAQGPOGAOGPLGIA-(GPP)$_5$-GPC-NH_2
44	GPC-(GPP)$_5$-GLAGQRGIVGLOGQRGERGFOGLOGPS-(GPP)$_5$-GPC-NH_2	44	GPC-(GPP)$_5$-GAOGPLGIAGITGARGLAGPOGMOGPR-(GPP)$_5$-GPC-NH_2
45	GPC-(GPP)$_5$-GFOGLOGPSGEOGKQGAOGASGDRGPO-(GPP)$_5$-GPC-NH_2	45	GPC-(GPP)$_5$-GPOGMOGPRGSOGPQGVKGESGKOGAN-(GPP)$_5$-GPC-NH_2

46	GPC-(GPP)$_5$-GASGDRGPOGPVGPOGLTGPAGEOGRE-(GPP)$_5$-GPC-NH$_2$	46	GPC-(GPP)$_5$-GESGKOGANGLSGERGPOGPQGLOGLA-(GPP)$_5$-GPC-NH$_2$
47	GPC-(GPP)$_5$-GPAGEOGREGSOGADGPOGRDGAAGVK-(GPP)$_5$-GPC-NH$_2$	47	GPC-(GPP)$_5$-GPQGLOGLAGTAGEOGRDGNOGSDGLO-(GPP)$_5$-GPC-NH$_2$
48	GPC-(GPP)$_5$-GRDGAAGVKGDRGETGAVGAOGAOGPO-(GPP)$_5$-GPC-NH$_2$	48	GPC-(GPP)$_5$-GNOGSDGLOGRDGSOGGKGDRGENGSO-(GPP)$_5$-GPC-NH$_2$
49	GPC-(GPP)$_5$-GAOGAOGPOGSOGPAGPTGKQGDRGEA-(GPP)$_5$-GPC-NH$_2$	49	GPC-(GPP)$_5$-GDRGENGSOGAOGAOGHOGPOGPVGPA-(GPP)$_5$-GPC-NH$_2$
50	GPC-(GPP)$_5$-GKQGDRGEAGAQGPMGPSGPAGARGIQ-(GPP)$_5$-GPC-NH$_2$	50	GPC-(GPP)$_5$-GPOGPVGPAGKSGDRGESGPAGPAGAO-(GPP)$_5$-GPC-NH$_2$
51	GPC-(GPP)$_5$-GPAGARGIQGPQGPRGDKGEAGEOGER-(GPP)$_5$-GPC-NH$_2$	51	GPC-(GPP)$_5$-GPAGPAGAOGPAGSRGAOGPQGPRGDK-(GPP)$_5$-GPC-NH$_2$
52	GPC-(GPP)$_5$-GEAGEOGERGLKGHRGFTGLQGLOGPO-(GPP)$_5$-GPC-NH$_2$	52	GPC-(GPP)$_5$-GPQGPRGDKGETGERGAAGIKGHRGFO-(GPP)$_5$-GPC-NH$_2$
53	GPC-(GPP)$_5$-GLQGLOGPOGPSGDQGASGPAGPSGPR-(GPP)$_5$-GPC-NH$_2$	53	GPC-(GPP)$_5$-GIKGHRGFOGNOGAOGSOGPAGQQGAI-(GPP)$_5$-GPC-NH$_2$
54	GPC-(GPP)$_5$-GPAGPSGPRGPOGPVGPSGKDGANGIO-(GPP)$_5$-GPC-NH$_2$	54	GPC-(GPP)$_5$-GPAGQQGAIGSOGPAGPRGPVGPSGPO-(GPP)$_5$-GPC-NH$_2$
55	GPC-(GPP)$_5$-GKDGANGIOGPIGPOGPRGRSGETGPA-(GPP)$_5$-GPC-NH$_2$	55	GPC-(GPP)$_5$-GPVGPSGPOGKDGTSGHOGPIGPOGPR-(GPP)$_5$-GPC-NH$_2$
56	GPC-(GPP)$_5$-GPRGRSGETGPAGPOGNOGPOGPOGPO-(GPP)$_5$-GPC-NH$_2$	56	GPC-(GPP)$_5$-GPIGPOGPRGNRGERGSEGSOGHOGQO-(GPP)$_5$-GPC-NH$_2$
57	GPC-(GPP)$_{10}$-GPC-NH$_2$	57	GPC-(GPP)$_5$-GERGSEGSOGHOGQOGPOGPOGAO-(GPP)$_5$-GPC-NH$_2$
58	GCO-(GPO)$_{10}$-GCOG-NH$_2$	58	GPC-(GPP)$_{10}$-GPC-NH$_2$
59	GPC-(GPP)$_5$-GFOGER-(GPP)$_5$-GPC-NH$_2$	59	GCO-(GPO)$_{10}$-GCOG-NH$_2$
		60	GPC-(GPP)$_5$-GFOGER-(GPP)$_5$-GPC-NH$_2$
